# The 30-day hospital readmission and mortality after surgery in colorectal cancer patients

**DOI:** 10.1186/s12876-022-02516-2

**Published:** 2022-10-14

**Authors:** Mesnad S. Alyabsi, Anwar H. Alqarni, Latifah M. Almutairi, Mohammed A. Algarni, Kanan M. Alshammari, Adel Almutairi, Nahar A. Alselaim

**Affiliations:** 1 Population Health Research Section, King Abdullah International Medical Research Center, PO Box 22490, Riyadh, 11426 Saudi Arabia; 2 Population Health Research Section, King Abdul Aziz Medical City, King Abdullah International Medical Research Center, Jeddah, 22384 Saudi Arabia; 3 Oncology Department, Ministry of National Guard - Health Affairs, Riyadh, Saudi Arabia; 4 King Saud Bin Abdulaziz University for Health Sciences, PO Box 22490, Riyadh, 11426 Saudi Arabia; 5 King Abdullah International Medical Research Center, PO Box 22490, Riyadh, 11426 Saudi Arabia; 6 Health and Rehabilitation Sciences College, Princess Noura Bint Abdul Rahman University, Riyadh, Saudi Arabia; 7 King Abdullah International Medical Research Center, Mail Code 1515, P.O. Box 3660, Riyadh, 11481 Saudi Arabia

**Keywords:** Colorectal cancer, Patient readmission, Cancer registry, Surgery

## Abstract

**Purpose:**

Hospital readmissions in the first weeks following surgery are common, expensive, and associated with increased mortality among colorectal cancer patients. This study is designed to assess the 30-day hospital readmission after colorectal cancer surgery and evaluate the risk factors that affect hospital readmission.

**Methods:**

The study uses data from the Ministry of National Guard-Health Affairs Cancer Registry. All colorectal cancer patients who underwent colorectal cancer surgery between January 1, 2016, and November 31, 2021, were investigated. Factors examined were age, gender, marital status, Body Mass Index, Charlson Comorbidity Index, chemotherapy, radiotherapy, tumor stage, grade, site, surgical approach, length of stay, and discharge location. Kaplan–Meier curves were constructed to assess survival rates between readmitted and non-readmitted patients, and logistic regressions were performed to assess predictors of readmission.

**Results:**

A total of 356 patients underwent tumor resection and 49 patients were readmitted within 30-day of index discharge. The most common reasons for hospital readmissions were gastrointestinal (22.45%), urinary tract infection (16.33%), and surgical site infection (12.24%). In the multivariable analysis, females were 89% more likely to be readmitted compared to males (odds ratio 1.89, 95% confidence intervals 1.00–3.58). Patients with distant metastatic tumors have higher odds of readmission (odds ratio 4.52, 95% confidence intervals 1.39–14.71) compared to patients with localized disease.

**Conclusions:**

Colorectal cancer readmission is more common in patients with metastatic disease. Strategies to reduce readmission include planned transition to outpatient care, especially among patients with a high risk of readmission.

**Supplementary Information:**

The online version contains supplementary material available at 10.1186/s12876-022-02516-2.

## Introduction

Colorectal cancer (CRC) is the most diagnosed cancer in Saudi males and the third most common cancer in females, with a 5-year observed survival rate of 52% [1,2]. Given the improved outcomes associated with the surgical resection of CRC, surgery remains the principal treatment modality for patients diagnosed with CRC [3]. Nonetheless, surgical outcomes including hospital readmission during the first weeks after surgery are prevalent, costly, and implicated with increased mortality [4–6]. Patients with cancer who have been discharged from hospitals have a readmission rate of up to 27% [7] and constitute a healthcare burden associated with morbidity and mortality [4]. No prior studies assessed the CRC rate of hospital readmissions and the risk factors associated with readmission among the Saudi population.

International studies, however, have evaluated CRC surgical outcomes including the 30-day hospital readmission and its predictors. Several reasons were associated with readmission including age, gender, race/ethnicity, type of health insurance, socioeconomic status (SES), tumor stage, comorbidities, procedure type, surgical approach (open or laparoscopic), length of hospital stay (LOS), surgical complications, non-home discharge, blood transfusion, and stoma creation [8]. Whether the rate and predictors of CRC readmissions are the same in the Saudi population remains unknown. This is vital given the poor characteristics of CRC in the Saudi population such as late-stage at diagnosis and low survival [9].

In Saudi Arabia, the total number of surgeries during the year 2020 was 398,188 and 46,831 of them were implemented at the Ministry of National Guard- Health Affairs (MNG-HA) [10]. More than 80% of the CRC patients at the MNG-HA are treated surgically [11], and hence elucidating the surgical outcomes of these patients is crucial. Similar to the US Veteran Affairs (VA) health system, the MNG-HA is a health system that covers eligible members in all regions of Saudi Arabia. Moreover, at the national level, Saudi Arabia has developed 12 strategic programs including a health sector transformation program that is mainly focusing on improving the population's health through efficient use of resources.

The present study aims to assess the 30-day hospital readmission after index colorectal cancer surgery and evaluates the risk factors that impact hospital readmission. We principally focused on the impact of demographic variables, tumor characteristics including the stage at diagnosis and LOS. We also investigated the reasons for hospital readmission. Identifying the rate of readmission and its predictors will contribute to the development of interventions that should reduce readmission, hospitalization costs and improve cancer patient outcomes.

## Materials and methods

### Data sources

The current study is a retrospective cohort study that uses data from the MNG-HA Cancer Registry linked with BESTCare, a medical record database within the MNG-HA system. The cancer registry contains information on patients’ demographics, clinical variables including tumor primary site, the stage at the time of diagnosis, and tumor grade for all patients who were diagnosed and treated at MNG-HA in Riyadh, Saudi Arabia. This study was approved by King Abdullah International Medical Research Center (NRC21R/432/10).

### Study population

All patients with histopathologically confirmed CRC diagnosis between January 1, 2016, and November 30, 2021, and ≥ 18 years old at the time of diagnosis among the MNG-HA population were included in the study. The MNG-HA population consists of military service members and their dependents, the civilian workforce, and healthcare students from the MNG-HA healthcare system. The population of more than one million individuals is served by tertiary care hospitals and four main primary and secondary care clinics.

### Study variables

#### Patient and tumor characteristics

Patient demographics were retrieved from the BESTCare system, including age at diagnosis, gender, marital status, and body mass index (BMI). Additional clinical variables such as comorbidities, chemotherapy, and radiotherapy were all retrieved. Inpatient records were used to gather surgical information such as type of surgery, LOS, surgery approach, operation duration, discharge location, admission, and discharge information.

Detailed tumor characteristics that were retrieved from the cancer registry include tumor site, topography, morphology, grade, and stage. The anatomic tumor location was categorized according to the International Classification of Diseases (ICD) for Oncology-third edition topography as follows: right colon (i.e., cecum, ascending colon, hepatic flexure of the colon, and transverse colon), left colon (i.e., splenic flexure of the colon, descending colon, and sigmoid), rectum (rectosigmoid junction and rectum) and colon not otherwise specified (NOS).

#### Outcome variables

CRC surgery was defined as the resection of the primary tumor with or without stoma creation within 1 month before or 12 months after CRC diagnosis. The 30-day readmission was defined as a hospitalization that occurred within 30 days of index discharge. Readmissions were identified using the inpatient records through admission and discharge dates. We also assessed overall survival in the study population. Follow-up time started from the index discharge date until the date of death, date of the last contact, or when the follow-up ended on January 31, 2022.

### Statistical analysis

Categorical variables were assessed using the chi-square test and Fisher exact test. The student t-test was used for comparing means, while Wilcoxon tests for comparing medians. Penalized logistic regression models with Firth correction were used to estimate the univariate and multivariable associations between readmission and covariates [1,2]. Forward elimination was used during the multivariable analysis with a criterion for entering variable to the model at *P* ≤ 0.15. These variables were gender, marital status, stage at diagnosis, and discharge location. We compared the death rate between readmitted and non-readmitted patients and generated the survival curves using the Kaplan–Meier method. The differences in survival estimates were compared using the log-rank test. All statistical tests were 2-sided, and findings were considered statistically significant at *P* ≤ 0.05. Data were analyzed using SAS statistical software version 9.4 (SAS Institute Inc. Cary, NC).

## Results

**Fig. 1:**
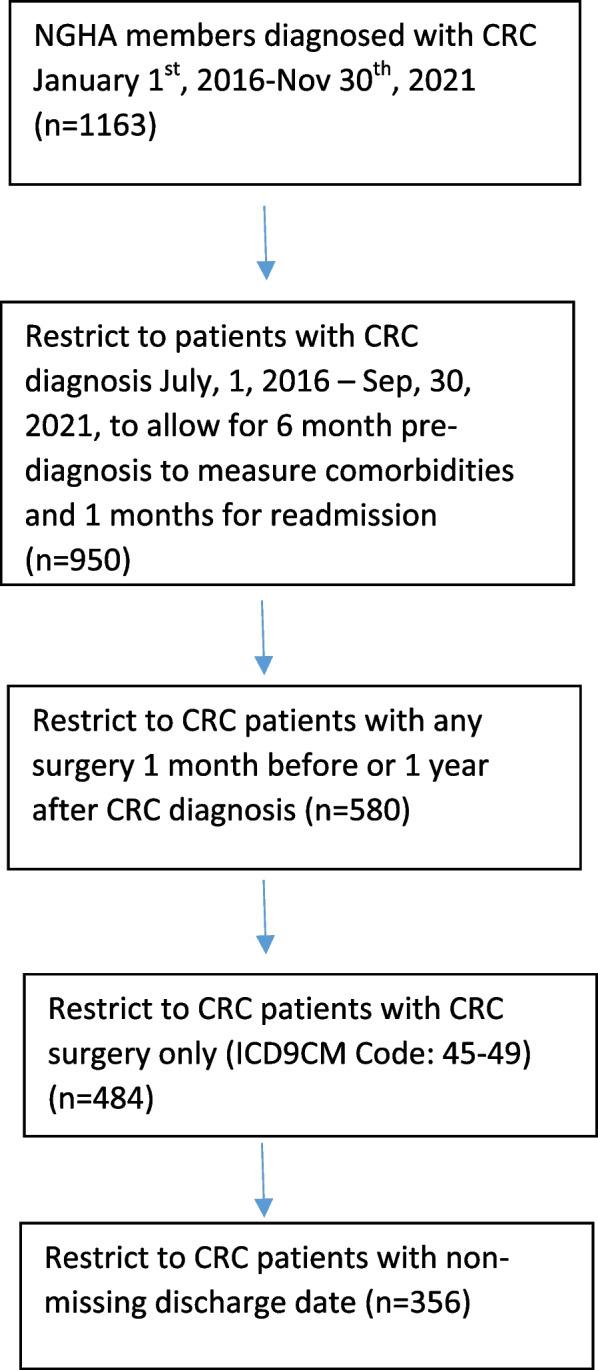
Eligibility criteria for the study population

Figure 1 shows the eligibility criteria for the study population. In the present study, 356 patients were included, and 49 (13.76%) of them were readmitted, while the remaining were non-readmitted patients (Table 1). The average age was 60.79 years with almost 60% of the sample being males and more than 78% were married. The average BMI was 27.28 kg/m^2^ and nearly 70% of the patients did not have any comorbid conditions. Compared to the readmitted patients, non-readmitted individuals were more likely to be male and presented with regional stage at diagnosis. There was a significant association between stage at diagnosis and hospital readmission (*P* value = 0.03).

**Table 1:** Patient characteristics by 30-days readmission status, NGHA 2016–2021 (N = 356)

	Readmitted		Non-readmitted		Total	*P*
	N	% or SD	N	% or SD	N, % or SD	
Overall	49	13.76	307	86.23	356	
Age (mean, SD)	62.75	13.40	60.48	13.83	60.79 (13.77)	0.28
Gender						
Male	24	48.98	189	61.56	213 (59.83)	0.09
Female	25	51.02	118	38.44	143 (40.17)	
Marital status						0.43
Single	1	2.04	16	5.21	17 (4.78)	
Married	43	87.76	236	76.87	279 (78.37)	
Divorces/widowed	1	2.04	20	6.51	21 (5.90)	
Unknown	4	8.16	35	11.40	39 (10.96)	
Body mass index (kg/m^2^)	26.75	6.36	27.36	5.71	27.28 (5.80)	0.53
Charlson comorbidity index						0.68
0	36	73.47	210	68.40	246 (69.10)	
1	8	16.33	67	21.82	75 (21.07)	
> 1	5	10.20	30	9.77	35 (9.83)	
Stage at diagnosis						
Distant metastasis	17	34.69	53	17.26	70 (19.66)	0.03
Regional	27	55.10	195	63.52	222 (62.36)	
Localized	4	8.16	51	16.61	55 (15.45)	
Missing	1	2.04	8	2.61	9 (2.53)	
Pathological grading						
Well differentiated	1	2.04	6	1.95	7 (1.97)	0.44
Moderately differentiated	41	83.67	274	89.25	315 (88.48)	
Poorly differentiated	5	10.20	16	5.21	21 (5.90)	
Unknown	2	4.08	11	3.58	13 (3.65)	
Tumor morphology						
Adenocarcinoma (AC), NOS	43	87.76	279	90.88	322 (90.45)	0.46
Mucinous AC	2	4.08	6	1.95	8 (2.25)	
Others	4	8.16	22	7.17	26 (7.30)	
Tumor site						
Right colon	6	12.24	53	17.26	59 (10.57)	0.56
Left colon	20	40.82	125	40.72	145 (40.73)	
Colon-nonspecified	12	24.49	82	26.71	94 (26.40)	
Rectum	11	22.45	47	15.31	58 (16.29)	
Chemotherapy						
Yes	7	14.29	31	10.10	38 (10.67)	0.38
No	42	85.71	276	89.90	318 (89.33)	
Radiotherapy						
Yes	19	38.78	90	29.32	109 (30.62)	0.18
No	30	61.22	217	70.68	247 (69.38)	

Table 2 displays the characteristics of the index surgery stratified by readmission status. The majority of the patients have a tumor located in the colon (83.71%) and the rest were in the rectum. Whereas 69.70% of the patients underwent open surgery, only 30.40% underwent laparoscopic surgery. Most patients had elective surgery (84.55%) and were discharged to the post-anesthesia care unit (82.87%). Furthermore, reasons for hospitalization after index discharge are reported in Table 3 and Additional file 1: Appendix 1. The recorded reasons were gastrointestinal (18.42% colon vs. 36.36% rectum), urinary tract infection (21.05% colon vs. 0% rectum), surgical site infection (13.16% colon vs. 9.09% rectum) and stoma related (2.63% colon vs. 27.27% rectum). Almost a quarter of readmission were due to gastrointestinal causes such as anastomotic leak, bowel obstruction, perforated tumor and rectal bleeding/discharge. Nevertheless, none of the differences were significant.

**Table 2:** Index surgery characteristics by 30-days readmission status, NGHA 2016–2021 (N = 356)

	Readmitted		Non-readmitted		Total	P
	N	% or SD	N	% or SD	N, % or SD	
Tumor location						
Colon	38	77.55	260	54.69	298 (83.71)	0.20
Rectum	11	22.45	47	15.31	58 (16.29)	
Surgery approach						
Laparoscopic	13	26.53	95	30.94	108 (30.43)	0.53
Open	36	73.47	212	69.06	248 (69.66)	
Surgery type						
Elective	43	87.76	258	84.04	301 (84.55)	0.84
Emergency	5	10.20	34	11.07	39 (10.96)	
Other	1	2.04	15	4.89	16 (4.49)	
LOS (median, IQR)	10.0	9.0	9.0	8.0	9.0 (8.0)	0.61
Discharge location						
Central Post Anesthesia Care Unit	38	77.55	257	83.71	295 (82.87)	0.05
Intensive Care Unit	6	12.24	41	13.36	47 (13.20)	
Other	5	10.20	9	2.93	14 (3.93)	
Operation time (minutes)	205.90	131.65	234.11	101.75	230.22 (106.60)	0.15

**Table 3:** Reasons for 30-day hospital readmission after surgery (n = 49)

Reasons for readmission	Rectum N (%) N = 11	Colon N (%) N = 38	Total N (%) N = 49	*P*
Gastrointestinal	4 (36.36)	7 (18.42)	11 (22.45)	0.20
Urinary tract infection	0 (0)	8 (21.05)	8 (16.33)	0.21
Surgical site infection	1 (9.09)	5 (13.16)	6 (12.24)	0.71
Stoma related	3 (27.27)	1 (2.63)	4 (8.16)	0.08
Cardiovascular	0 (0)	2 (5.26)	2 (4.08)	1.0
Others	3 (27.27)	15 (39.47)	18 (36.73)	0.45

In the univariate analysis, there was a positive association between age and readmission and females had higher odds of readmission than males, but both did not reach a significance level (Table 4). On the contrary, compared to patients diagnosed with a localized tumor, those diagnosed with distant metastatic disease have 4.09 higher odds of readmission (odds ratio 4.09, 95% confidence intervals 1.29–12.98). Likewise, patients discharged to locations other than post-anesthesia care or ICU have 3.75 higher odds of readmission (odds ratio 3.75, 95% confidence intervals 1.19–11.80).

**Table 4:** Univariate and multivariable analysis of risk factors for 30-day readmission, NGHA 2016–2021

	Univariate analysis		Multivariate analysis	
	OR	95% CI	OR	95% CI
Age				
≤ 40	1.0	1.0		
41–50	1.08	(0.24, 4.90)		
51–60	1.84	(0.49, 6.84)		
61–70	1.65	(0.44, 6.18)		
≥ 71	1.38	(0.36, 5.26)		
Gender				
Male	1.0	1.0		
Female	1.67	(0.91,3.05)		
Marital status				
Single	1.0	1.0		
Married	2.92	(0.38, 22.56)		
Divorces/widowed	0.80	(0.05, 13.81)		
Unknown	1.82	(0.19, 17.69)		
Body mass index (BMI)				
Under weight	2.22	(0.63, 7.86)		
Healthy weight	1.0	1.0		
Overweight	0.55	(0.25, 1.19)		
Obese	0.71	(0.33, 1.51)		
CCI				
0	1.0	1.0		
1	0.69	(0.31, 1.57)		
> 1	0.97	(0.35, 2.67)		
Stage at diagnosis				
Localized	1.0	1.0	1.0	1.0
Regional	1.76	(0.59, 5.27)	1.59	(0.55, 4.58)
Distant metastasis	4.09	**(1.29, 12.98)**	**3.86**	**(1.26,11.85)**
Missing	1.59	(0.16, 16.13)	2.19	(0.27,17.45)
Pathological grading				
Well differentiated	1.0	1.0		
Moderately diferentiated	0.89	(0.10, 7.65)		
Poorly differentiated	1.88	(0.18, 19.53)		
Unknown	1.09	(0.08, 14.66)		
Tumor morphology				
Adenocarcinoma (AC), NOS	1.0	1.0		
Mucinous AC	2.16	(0.42, 11.06)		
Others	1.17	(0.39, 3.59)		
Tumor site				
Right colon	1.0	1.0		
Left colon	1.41	(0.54, 3.72)		
Colon-nonspecified	1.29	(0.46, 3.65)		
Rectum	2.06	(0.71, 6.02)		
Chemotherapy				
Yes	1.0	1.0		
No	0.67	(0.28, 1.62)		
Radiotherapy				
Yes	1.0	1.0		
No	0.65	(0.35, 1.22)		
Surgery approach				
Laparoscopic	1.0	1.0		
Open	1.24	(0.63, 2.45)		
Surgery type				
Elective	1.0	1.0		
Emergency	0.88	(0.33, 2.38)		
Other	0.4	(0.05, 3.10)		
LOS				
≤ 9 Days	1.0	1.0		
> 9 Days	1.29	(0.71, 2.37)		
Discharge location				
Central Post Anesthesia Care Unit	1.0	1.0	1.0	1.0
Intensive Care Unit	0.98	(0.39, 2.49)	1.0	(0.40, 2.48)
Other	3.75	**(1.19, 11.80)**	4.19	**(1.31, 13.42)**

Bold values indicate significant results at* p*-value = 0.05

**Fig. 2:**
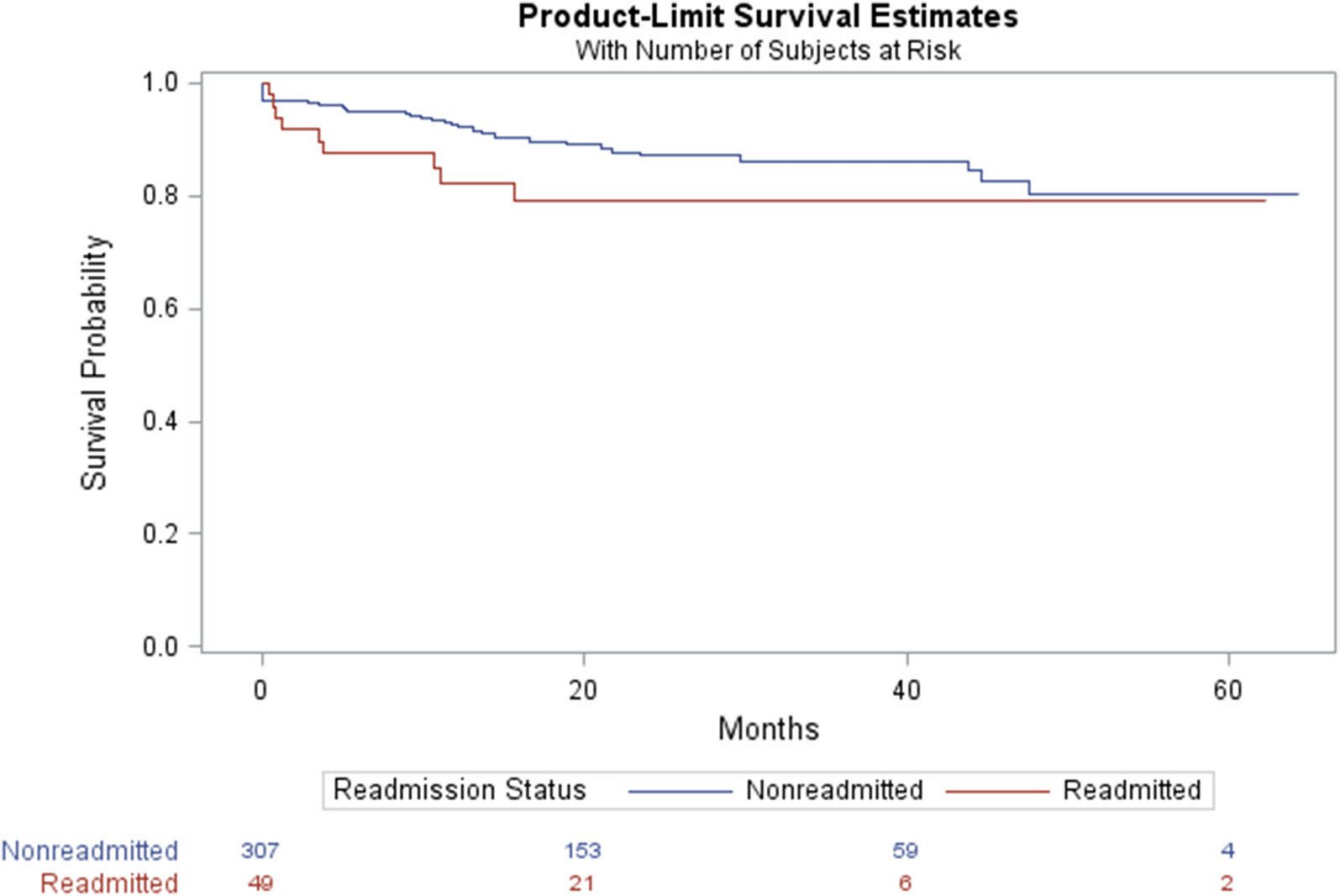
Kaplan–Meier analysis of overall survival of CRC patients after surgery

In the multivariable analysis, both patients diagnosed with distant metastatic tumors and those discharged to locations other than post-anesthesia care or ICU have higher odds of readmission (Table 4). Finally, for the mortality outcome, there is no statistically significant difference in the survivability amongst those readmitted and those not readmitted (*P* = 0.0581) (Fig. 2).

## Discussion

In this retrospective population-based cohort study, we found that 13.76% of CRC patients were readmitted within 30-day of index hospitalization, a finding that is slightly higher than previous studies (9–11%) [4,12–17]. The majority of recorded reasons for hospital readmissions were gastrointestinal (22.45%), urinary tract infection (16.33%), and surgical site infection (12.24%). Almost one-quarter of metastatic patients were readmitted within 30 day of index hospitalization.

While previous studies found that 22% to 27% of metastatic CRC patients were readmitted [18–21,24], we observed that 24.30% (17/70) of our metastatic patients were readmitted. In the multivariable analysis, metastatic patients had 3.86 higher odds of 30-day hospital readmission compared to those with localized disease, similar to previous findings [22,23]. Several studies from Saudi Arabia have shown that a sizable percentage of Saudi CRC patients were diagnosed at an advanced stage [2,9], a risk factor that has been shown to increase readmission in our population. Moreover, we found that patients discharged to locations other than post anesthesia care unit were more likely to be readmitted.

Taken together, hospitalized patients with metastatic disease should be counseled before discharge, for example through outpatient transition [24], to reduce hospital readmission. Alternatively, down-staging efforts through an early stage at diagnosis, as secondary public health prevention, will indirectly reduce the rate of hospital readmission [2,9].

Similar to some but not all prior research, we found no association between readmission and comorbidities, LOS, or surgical approach. In a population-based study that assessed readmission rates in the VA population, the authors found no significant association between the aforementioned factors on the rate of readmission [25]. On the contrary, some other studies found a positive association with comorbidities, LOS, and open surgery. Notably, our population has longer index hospitalization compared to other populations [4,5,13,26]. It is possible that lack of association is due to the small observed number in the readmitted patients particularly those with > one comorbidity score.

The results of the present study should be interpreted within the scope of the following limitations. First, the reported results should be generalized to the MNG-HA population or a similar population. Second, SES is a factor that was affecting readmission in some previous studies which were not accounted for in the current study. Nonetheless, given that the MNG-HA population has equitable access to care and that all members are employed by the system, the SES effect is modest. Third, among patients with metastatic disease, we were not able to distinguish between primary tumor resection from metastatic resection. Fourth, given the positive volume-outcome relationship in CRC patients, adjusting for such factors could have improved the finding of the present study. Lastly, some of our admitted patients (n = 128) were missing discharge date and were excluded from analysis. The characteristics of these patients, nonetheless, were similar to our study population (Additional file 1: Appendix 2).

The knowledge of the rate and factors for hospital readmission in CRC patients has a significant impact on patients and the healthcare system. Given the identified factors, implementing strategies that may reduce readmission rates is needed. For instance, the adoption of minimally invasive surgery (e.g. laparoscopic procedure) could potentially contribute to lower hospitalization after major surgeries such as CRC. Additionally, post-discharge strategies include shorter outpatient follow-up time, nursing or home health care visits, and making a nursing/ educator home phone call. Many other modalities may be studied and implemented.

## Conclusion

CRC readmission is common, especially in patients with metastatic disease. Strategies to reduce readmission include planned transition to outpatient care, especially among patients with a high risk of readmission.

## Supplementary Information

**Additional file 1.** This file includes a table of the reasons for 30-day hospital readmission after surgery, in addition to another table representing a comparison between the study population and patients with missing discharge date.

## Data Availability

The datasets generated and/or analysed during the current study are available from the corresponding author upon a reasonable request. The data are not publicly available due to privacy and ethical concerns by the Ethics Committee of the KAIMRC.

## Abbreviations

CRC: Colorectal cancer
MNG-HA: Ministry of National Guard-Health Affairs
ICD: International classification of diseases
OS: Overall survival
SSI: Surgical site infections
CCI: Charlson comorbidity index
BMI: Body mass index
