# Supplementary material for: The 30-day hospital readmission and mortality after surgery in colorectal cancer patients
Source: BMC Gastroenterol. 2022 Oct 14;22:434. doi: 10.1186/s12876-022-02516-2 (PMC9563783; doi:10.1186/s12876-022-02516-2)
Supplement: Supplementary file 1 — Additional file 1. This file includes a table of the reasons for 30-day hospital readmission after surgery, in addition to another table representing a comparison between the study population and patients with missing discharge date. [file 12876_2022_2516_MOESM1_ESM.docx]

| **Appendix 1.** **Reasons for 30-day hospital readmission after surgery (n=49)** | | | | |
| --- | --- | --- | --- | --- |
| **Reasons for readmission** | **Surgery approach** | | **Surgery type** | |
|  | **Open** | **Laparoscopic** | **Elective** | **Emergency** |
| **Gastrointestinal** | 5 | 6 | 10 | 1 |
|  |  |  |  |  |
| **Urinary tract infection** | 7 | 1 | 8 | 0 |
|  |  |  |  |  |
| **Surgical site infection** | 4 | 2 | 5 | 1 |
|  |  |  |  |  |
| **Stoma related** | 3 | 1 | 3 | 1 |
|  |  |  |  |  |
| **Cardiovascular** | 2 | 0 | 1 | 1 |
|  |  |  |  |  |
| **Others** | 15 | 3 | 16 | 2 |

**Additional file 1.**

This file includes a table of the reasons for 30-day hospital readmission after surgery, in addition to another table representing a comparison between the study population and patients with missing discharge date.

| **Appendix 2.** **Comparison between the study population and patients with missing discharge date** | | | |
| --- | --- | --- | --- |
|  | Study population (n=356) | Patients with missing discharge date (n=128) |  |
|  | N (%) | N (%) | P |
| **Age (mean, SD)** | 60.79 (13.77) | 60.20 (12.60) | 0.65 |
| **Gender,** Male | 213 (59.83) | 81 (63.29) | 0.39 |
| **Marital status** |  |  |  |
| Single | 17 (4.78) | 8 (6.25) | 0.80 |
| Married | 279 (78.37) | 103 (80.47) |  |
| Divorces/widowed | 21 (5.90) | 7 (5.47) |  |
| Unknown | 39 (10.96) | 10 (7.81) |  |
| **BMI (kg/m^2^)** | 27.28 (5.80) | 27.81 (7.51) | 0.47 |
| **CCI** |  |  | 0.51 |
| 0 | 246 (69.10) | 83 (64.84) |  |
| 1 | 75 (21.07) | 28 (21.87) |  |
| >1 | 35 (9.83) | 17 (13.28) |  |
| **Stage at diagnosis** |  |  | 0.33 |
| Distant metastasis | 70 (19.66) | 25 (19.53) |  |
| Regional | 222 (62.36) | 71 (55.47) |  |
| Localized | 55 (15.45) | 30 (23.44) |  |
| Missing | 9 (2.53) | 2 (1.56) |  |
| **Pathological grading** |  |  | 0.90 |
| Well differentiated | 7 (1.97) | 3 (2.34) |  |
| Moder. differentiated | 315 (88.48) | 114 (89.06) |  |
| Poorly differentiated | 21 (5.90) | 8 (6.25) |  |
| Unknown | 13 (3.65) | 3 (2.34) |  |
| **Surgery type** |  |  | 0.55 |
| Elective | 301 (84.55) | 111 (86.72) |  |
| **Discharge location** |  |  |  |
| Central Unit | 295 (82.87) | 105 (82.03) | 0.75 |
| ICU | 47 (13.20) | 16 (12.50) |  |
| Other | 14 (3.93) | 7 (5.47) |  |
| **Operation time (min)** | 230.22 (106.60) | 228.5 (143.0) | 0.47 |
